# A Neural Network Approach for Chinese Sports Tourism Demand Based on Knowledge Discovery

**DOI:** 10.1155/2022/9400742

**Published:** 2022-04-04

**Authors:** Libin Qi, Yaohan Tang

**Affiliations:** ^1^Sports College, Hunan University of Finance and Economics, Changsha, China; ^2^Sports College, Hunan Agricultural University, Changsha, China

## Abstract

With the vigorous development of the Chinese economy and people's pursuit of quality, sports activities of people pursuit are no longer limited to simple physical exercise, but a way that pursues higher-quality sports tourism. As a new industry, it cannot guarantee that sports tourism will be accepted by all people, and it will be limited by geographical, economic, time, and other conditions. The participation number of Chinese sports tourism is more concerned by organizers or operators. Predicting the participation number of sports tourism based on the knowledge discovery method is meaningful and economical work. In this paper, a variety of sports tourism data are classified by clustering method, and the categories with similar characteristics are classified. Then, the convolution and long short-term memory hybrid neural network are used to extract the spatial and temporal information of sports tourism characteristics, which completes the prediction of Chinese sports tourism categories. The research results show that the clustering method has high accuracy for the classification of sports tourism categories, and the weights occupied by the categories are relatively uniform. The ConvLSTM neural network also has obvious advantages in predicting Chinese sports tourism methods. The largest error is only 2.89%, and the correlation coefficient also reaches 0.98, which is enough to be trusted for the prediction of Chinese sports tourism categories.

## 1. Introduction

With the rapid development of the Chinese economy and the continuous improvement of national quality, people are more and more aware of the importance of sports [1]. More people will participate in physical exercise, not only will they actively participate in physical exercise, but they will also actively watch sports events, which greatly stimulates the continuous development of the sports wave in China and even the world. However, in recent years, with the rapid development of the national economy and the changes in market demand brought about by the differentiation of income classes, the combination of tourism and sports has developed rapidly [2]. This way not only makes people satisfy their desire to travel but also guarantees their health, which is a popular way. Sports tourism is a form of tourism activities that people can participate in or watch sports events for the purpose or take sports as the main content, such as dragon boat racing, Tujia hand dance, and Naadam conference [3]. Sports tourism can not only improve people's enthusiasm for exercising but also satisfy people's interest in appreciating natural scenery. Sports tourism can meet people's two needs at the same time, which promotes the development of the Chinese sports tourism industry [4].

Sports tourism is simply the combination of sports and tourism, and sports can be divided into watching events or participating in sports and sports cultural experience [5]. Sports tourism is a form of tourism activity in which people participate in and watch sports or take sports as the main content. In a broad sense, sports tourism is the sum of the relationship between the various physical entertainment, physical exercise, sports competition, sports rehabilitation, and sports cultural exchange activities that tourists engage in tourism and tourism destinations, sports tourism enterprises, and society [6, 7]. Sports tourism can be understood as the manager will use various sports activities to promote the physical and mental development of tourists. This is also to meet the various sports needs of tourists. Sports can enrich people's social and cultural activities, and it can achieve the purpose of promoting the material and spiritual civilization of society [8]. Since the Beijing Olympic Games, domestic sports tourism has developed rapidly. In recent years, the state has promulgated a series of policies to support the development of sports tourism. With the holding of various international and domestic competitions, people's enthusiasm for participating in sports tourism has increased quick boost [9].

In recent years, the sports tourism industry in China and around the world has developed rapidly, and many studies on the sports tourism industry have also been carried out in the current public research [10]. Chang et al. [11]used the SmartPLS method to study sports tourism and local residents support rate for sports tourism. It also studies the impact of tourism knowledge on the development of local sports tourism. Finally it examines the limitations of sports tourism. Zhu et al. [12] believe that the coastal leisure tourism industry has huge development space and opportunities, but the form of coastal tourism is significantly different from other forms of sports tourism. He used the RMP theoretical analysis method to study the development space and feasibility of the coastal sports tourism industry. At the same time, he studied the problems and challenges in the development of coastal sports tourism. This research can provide a certain reference value for the development of the coastal sports tourism industry. Machine learning methods can have better performance in the classification and prediction of natural areas. Yang et al. [13] have used the FPGA model to perform a correlation analysis on the two main factors affecting the development of the sports tourism industry, the local economic development, and the residents' support rate for sports tourism. At the same time, the collected resident information is predicted by multiple regressions, and the results show that these two independent variables have an important impact on the development of the sports tourism industry. Dickson et al. [14] analyzed the impact of the Vancouver Winter Olympics on promoting the development of sports tourism. He used the WAS method to study the impact of local sporting event heritage on residents' development of sports tourism, as well as the needs and desires of local residents and tourists for sporting event heritage. Li et al. [15] believe that the One Belt One Road strategy implemented in coastal areas has promoted the development of the coastal tourism industry. The combination of the coastal tourism industry and sports will also be favored by tourists. He analyzes the homogenization, vulgarization, and destructiveness of the form of maritime sports tourism, which will promote the development of sports tourism through the One Belt One Road strategy. Chen et al. [16] believe that there is an obvious nonequilibrium relationship between the oversupply of the sports tourism industry and the continuous rise in prices. He used the STCRPS model to integrate sports tourism, sports tourism projects, and consumers to make related predictions and used the Gaussian model and regression to make classification predictions. The results show that the Gaussian mixture model has better performance in predicting sports tourism. Kuchumov et al. [17] mainly study the definition of sports tourism and compare and analyze the definition of sports tourism with foreign researchers. He suggested that sports tourism should be defined by tourists who leave their hometowns to participate in sports tourism in different places. Han et al. [18] conducted research on the uneven distribution of rural sports tourism in the geographical space and regions and the low classification accuracy. He used the radius of gyration to divide the area and classified and predicted the position difference and spatial difference. Yue et al. [19] use the integrated interactive system CRMNST to assess the risk of the sports tourism industry. For the study of tourism, Mohsin et al. [20] conducted research on halal tourism and its impact on community residents. Therefore, a lot of scholars have done some work in this field. Meanwhile, some machine learning models including neural networks and big data techniques have been proposed and are widely used in various fields [21–23], in which these techniques can also be applied to sports tourism.

China is a country with vast land and abundant resources, and its natural scenery is quite different for different latitudes and longitudes, which has resulted in the rapid development of the tourism industry [24]. There are large differences in natural scenery caused by regional differences in China, and China has deep historical information, which has created the development of multicultural differences within China. People can appreciate and experience different natural scenery, which promotes the development of tourism in China [25]. Moreover, with the development of the Chinese national economy and the improvement of spiritual living standards, people have begun to pursue a high-quality life [26]. The sports industry has grown over the years and has reached a booming level. The results of sports and tourism are increasingly sought after by the nation [27]. However, Chinese people's pursuit of sports tourism is uneven, which leads to difficulties for sports tourism organizers. Tourism industry researchers in China and even in the world have done a lot of research on the sports tourism industry. How to predict the types of sports tourism in China and Chinese natural and cultural differences is a meaningful way to promote the development of the Chinese sports tourism industry [28]. This study employs clustering and neural network techniques to deal with data features in Chinese sports tourism. Clustering has been widely used in feature classification tasks and has shown good performance. Neural network technology is also widely used in nonlinear data processing by domestic and foreign scholars. Current research on sports tourism mainly uses classification methods for category classification without involving further prediction tasks. This paper firstly uses the clustering method to classify the characteristics of Chinese sports tourism tasks and then uses the neural network method to predict the development trend of sports tourism, which is a meaningful thing for the initiators of sports tourism.

This paper mainly forecasts the type and development trend of Chinese sports tourism, which is mainly divided into five parts. The first part introduces the development trend of sports tourism and the forecast development of the sports tourism industry. The second part mainly introduces the significance and necessity of knowledge discovery for Chinese sports tourism prediction. The third part mainly introduces the mathematical model used to predict the Chinese sports tourism industry. The fourth part evaluates knowledge discovery techniques in forecasting Chinese sports tourism from the perspective of prediction accuracy and error, and the fifth part is the conclusion of the article.

## 2. The Necessity and Data Sources of Knowledge Discovery for Forecasting China Sports Tourism Industry

### 2.1. The Significance of Knowledge Discovery Models for Predicting Sports Tourism

With the continuous development of the national economy and the improvement of people's needs for living standards, more and more people are participating in physical exercise and tourism [29, 30]. However, people are no longer simply doing tourism in the form of appreciation of the scenery but more and more to participate in some tourism projects with a sports nature. This way can not only meet the tourist purpose of enjoying the scenery but also realize the purpose of physical exercise. My country's sports tourism industry has also developed rapidly, which is mainly due to the diversified development of my country's customs [31]. However, diversified geographical differences have resulted in the uneven development of sports tourism, which has caused great difficulties for the organizers of sports tourism [32]. Then, there is the level of local economic development, which also seriously restricts the participation of the sports tourism industry. In order to organize the sports tourism industry with higher quality and achieve an economic win-win between participants and local organizers, it is necessary to make accurate forecasts for the Chinese sports tourism industry [33]. This work is very beneficial to the organization of the sports tourism industry. Through the introduction of the first part, it can be seen that people have made more predictions about the sports tourism industry at this stage. The development of data mining technology and knowledge discovery model provides great convenience for the prediction of the Chinese sports tourism industry [34]. Knowledge discovery is actually a method of data mining, which can find more correlations from a large number of data, which provides the possibility for the prediction of the sports tourism industry [35]. The knowledge discovery method can find data such as the level of local economic development and local natural resources and the participation rate of sports tourism activities in previous years [36]. The knowledge discovery model can accurately predict the participation and economic efficiency of the sports tourism industry. It can not only improve the support rate of local residents for the organization of the sports tourism industry but also allow various forms of activities to attract more tourism hobbies to participate. This is meant for increasing national participation as well as improving the level of the local economy.

### 2.2. The Data Sources for China Sports Tourism Forecast

This paper mainly aims at the uneven participation caused by the large natural regional differences in the Chinese sports tourism industry and uses the knowledge discovery model combined with the local humanistic and economic level to make accurate predictions for the development of the sports tourism industry. Knowledge discovery is a technique of data mining from a large number of data to discover more relevant data. This research first selects the local economic level, local sports tourism projects, participation in the sports tourism industry, residents support rate, and other data for data mining. Knowledge discovery technology can find highly relevant information from the data of economy, participation, and sports tourism form and then use neural network technology to make further predictions. The data select sports tourism projects carried out in Xishuangbanna tourist attractions to extract relevant information. The ultimate purpose of this paper is to use knowledge discovery methods to discover the participation of the Chinese sports tourism industry and the benefits it brings to the local economy. Therefore, firstly, the natural scenery resources, local economic level, residents' support rate survey, developable sports tourism projects, and participation in sports tourism projects in the development area of the Chinese sports tourism industry are collected as the input of the knowledge discovery model, and the relationship between them is calculated. The relationship is mined and classified, and finally, the data are used as the input of the neural network to predict the local sports tourism projects that can be developed in the next stage and predict the expected participation and economic benefits. This prediction method will not only increase people's participation in local sports tourism projects but also increase people's enthusiasm for sports tourism projects and have a profound impact on local economic benefits. The input data set of this paper is mainly the passenger participation volume of the Chinese sports tourism industry, the local tourism economic level, and the type of tourism projects, and the output is the passenger participation volume of future sports tourism projects. These data sets are divided into training, validation, and test sets Table 1.

## 3. The Knowledge Discovery Models and Intelligent Prediction Algorithms for Sports Tourism

### 3.1. The Introduction of Knowledge Discovery Model

**Figure 1:**
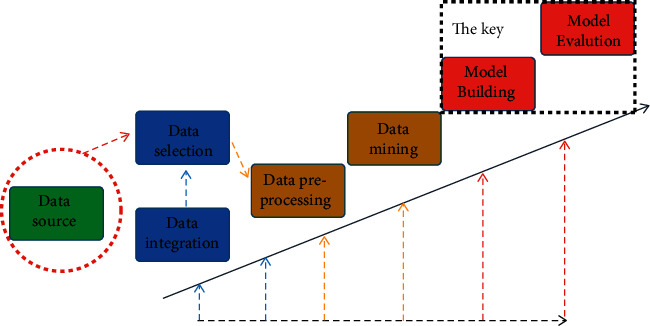
The process of knowledge discovery processing Chinese sports tourism data.

Knowledge discovery is actually another interpretation of data mining technology. It is a way of classifying and summarizing knowledge information from various sources of information according to different needs and different methods. The purpose of knowledge discovery is to shield the correlation information between the original data, extract meaningful and concise information from the original data, and report it directly to the user. This paper hopes to realize the prediction of participation in the Chinese sports tourism industry and the benefits to the local economy by means of knowledge discovery. However, it is extremely difficult to rely solely on local sports tourism organizers to discover the correlations between these complex data and guide the development of the sports tourism industry. Data mining technology just provides technical support for the prediction of the sports tourism industry. This model allows local natural resources, participation in the sports tourism industry, and local economic factors as the input of the model and then outputs the amount of data that the organizers are concerned about.  Figure 1 shows the process of knowledge discovery technology in the prediction of Chinese sports tourism data. First, it is necessary to collect some data sources, select and enhance the data, and process the data forms and data sources required by the clustering model. Then, these data sources need to be normalized and averaged at the feature level, so that the model can better balance the distribution of weights. Then, these data sources need to fully extract the correlation information between the data through the data mining model. Finally, by performing data prediction and data evaluation on the data processed by data mining technology, it will find the amount of data required by sports tourism organizers, and at the same time, this will have certain guiding significance for the development organizers of Chinese sports tourism [37]. This study first classifies the data collected about sports tourism in China in order to better progress the optimization problem with neural network techniques. For the first time, it utilizes the advantages of convolutional neural networks in processing spatial features to extract features from factors such as passenger participation in the sports tourism industry and local economy. Then, the advantage of the recurrent neural network in temporal feature extraction is used to effectively extract the temporal features of sports tourism, and finally, it maps the correlation relationship.

### 3.2. A Clustering Method for Chinese Sports Tourism Data

**Figure 2:**
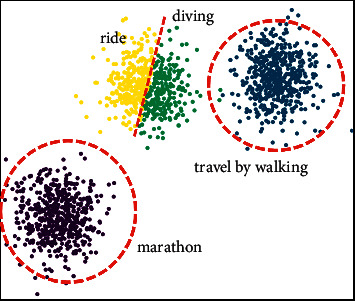
The schematic diagram of the application of clustering in the Chinese sports tourism industry.

Clustering is an important classification method in knowledge discovery technology, which is to classify different related data according to the distance relationship between data sources. The final effect is to divide the data with a strong correlation into one category, and the data with poor correlation between the data into different categories, and the farther the distance between the different categories of data, the better the effect. For the Chinese sports tourism industry, the sources of data are complex and extensive [38]. Before making predictions on these data, these data sources need to be clustered first. For the effect of clustering, there are mainly two forms of clustering methods based on distance and density. Since the data sources selected in this paper are the types of natural scenery in the sports tourism industry, the participation of sports tourism activities, and the support rate of residents, these data sources are more suitable for clustering processing based on distance. Therefore, this paper chooses the K-means clustering method to perform data mining processing on the data source of sports tourism.  Figure 2 shows the effect diagram of the clustering method applied in the Chinese sports tourism industry. It is only an effect diagram of clustering, not the result of this article. The original image comes from the website https://image.so.com. It can be seen from Figure 2 that the collected data sources can be divided into 4 different forms of categories by clustering, and the interfaces between their different categories are relatively obvious, and the data with strong correlation are divided into the same category.

Equation (1) shows the error function used by the clustering method, which uses the mean square error as the error function for the clustering backpropagation process.

(1) $$\begin{array}{c} J=\frac{1}{2}{\sum_{k=1}^{l}\sum_{r=1}^{o}\left(x_{k}-\chi_{r}\right)}^{2}. \end{array}$$

Equation (2) illustrates the objective function used in the clustering process, which is a convex function. The clustering process is the process of continuously minimizing the value of this function, and the gradient method is used.

(2) $$\begin{array}{c} \chi =\frac{1}{N_{K}}\sum_{r=1}^{n_{j}}x_{k}. \end{array}$$

Equation (3) illustrates the process of derivation of the objective function to solve the gradient. By continuously solving the minimum gradient of this function, the average value of the optimal cluster center is found.

(3) $$\begin{array}{c} \frac{\partial J}{\partial \chi_{J}}=-2\sum_{k=1}^{N_{j}}\left(x_{k}-\chi_{r}\right). \end{array}$$

The effect of clustering can be evaluated according to different distance functions to find the optimal weight and optimal clustering effect. Equation (4) shows the expression for the Euclidean distance, a measure of the distance between two points in space.

(4) $$\begin{array}{c} {\text{dict}}_{ed}=\sqrt{\sum_{k=1}^{m}{\left(x_{ik}-x_{jk}\right)}^{2}}. \end{array}$$

This is a method of finding the optimal weights through the limit method. Equation (5) shows the Chebyshev distance, an evaluation index of the clustering effect.

(5) $$\begin{array}{c} {\text{dict}}_{cd}=\underset{t⟶\infty }{\lim}{\left({\sum_{k=1}^{m}\left|x_{jk}-y_{ik}\right|}^{l}\right)}^{1/l}. \end{array}$$

Equation (6) shows the Minkowski distance evaluation index, which is a measure in Euclidean space.

(6) $$\begin{array}{c} {\text{dict}}_{\min d}={\sqrt[{p}]{\sum_{k=1}^{m}\left|x_{ik}-y_{jk}\right|}}^{p}. \end{array}$$

### 3.3. A Neural Network Approach for Chinese Sports Tourism Data Sources

After the collected data sources related to sports tourism are clustered by the knowledge discovery technology, the data sources will be in different categories according to a certain method. These different classes of data sources are then predicted by neural network methods. There is not only a certain spatial feature correlation between the data sources related to the sports tourism industry but also a certain time correlation between them. In this paper, the ConvLSTM neural network is selected to predict the demand for sports tourism in China. ConvLSTM neural network can not only handle time series like LSTM neural network technology, and it can also handle spatial features very well [39, 40]. This is due to the fact that the dot product operation of this neural network model becomes a convolution operation compared to the LSTM neural network method. Whether it is the prediction of spatial features or temporal features, the ConvLSTM neural network shows better performance compared to other neural network methods.

**Figure 3:**
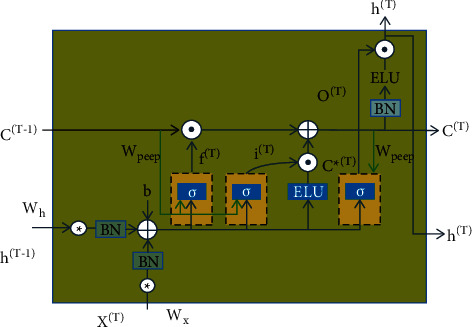
The prediction process of ConvLSTM for the Chinese sports tourism industry.

Figure 3 shows a schematic diagram of the workflow of the recurrent neural network. Its input gate will receive the output information of the CNN neural network and the historical state information of the previous state, which is a continuous process. This is because there is a strong temporal correlation in the Chinese sports tourism industry, so the recurrent neural network in Figure 3 is adopted.

The ConvLSTM neural network has a certain similarity with the LSTM network. Equation (7) shows the operation flow of the perceptron part of the neural network, which is also part of the forget gate. It is a partial selection of historical state information and a partial selection of input data and then outputs through a certain weight.

(7) $$\begin{array}{c} \alpha_{l}^{t}=\sum_{t=1}^{k}\omega_{il}x_{i}^{t}+\sum_{b=1}^{v}\omega_{ct}s_{b}^{t-1}. \end{array}$$

Equations (8) and (9) illustrate the state flow of the input gate, which comprehensively processes the input of the forget gate and the input data source of sports tourism and undergoes nonlinear processing of the activation function.

(8) $$\begin{array}{c} i_{t}=\sigma {\left(W_{xi}*x_{t}+W_{hi}*h_{t-1}+W_{ci}\circ C_{t-1}+b\right)}_{i}. \end{array}$$

(9) $$\begin{array}{c} f_{t}=\sigma \left(W_{xf}*x_{t}+W_{hf}*h_{t-1}+W_{cf}\circ C_{t-1}+b_{f}\right). \end{array}$$

It can refresh the variable by the following formula:

(10) $$\begin{array}{c} C_{t}=f_{t}\circ C_{t-1}+i_{t}\circ ELU\left(W_{xc}*x_{t}+W_{hc}*h_{t-1}+b_{c}\right). \end{array}$$

Equations (11) and (12) illustrate the operation flow of the output gate of the neural network. It will assign different weights to the historical state information and the processed input gate information, and then, some data sources are output through the output gate, and finally, feature mapping is performed through the nonlinear processing of the activation function.

(11) $$\begin{array}{c} o_{t}=\sigma \left(W_{xo}*x_{t}+W_{ho}*h_{t-1}+W_{co}\circ C_{t}+b_{o}\right), \end{array}$$

(12) $$\begin{array}{c} h_{t}=o_{t}\circ ELU\left(C_{t}\right). \end{array}$$

### 3.4. Preprocessing of Chinese Sports Tourism Data Sources

It can also be seen from Figure 1 that the selection of data sources and data preprocessing in the knowledge discovery process occupies a large part of the process, which shows that data preprocessing is a crucial process for data mining and neural network prediction. This is because the collected data sources have distinct differences in characteristics and magnitudes. For the data sources of the Chinese sports tourism industry, this preprocessing process is important, so there are obvious differences in the characteristics and data forms of data sources such as types of natural scenery and participation rates. The data preprocessing process needs to process the data related to the sports tourism industry into data that conforms to the same distribution and is in the same order of magnitude, which is beneficial for the uniform distribution of weights of the clustering method and the neural network method, which can make the prediction results and classification results more accurate. The learning rate of 0.001 and the number of filters of 64 were adopted to train the model in this paper, and the data set was divided into the training set, validation set, and test set. The model in this paper is trained on the TensorFlow platform and GPU, and the iteration step is 3000. The input data of this paper are the passenger flow of previous years, local economy, and sports tourism projects in sports tourism, and the output data are the passenger participation in sports tourism.

## 4. The Analysis and Discussion on Forecast Accuracy of Chinese Sports Tourism Industry

**Figure 4:**
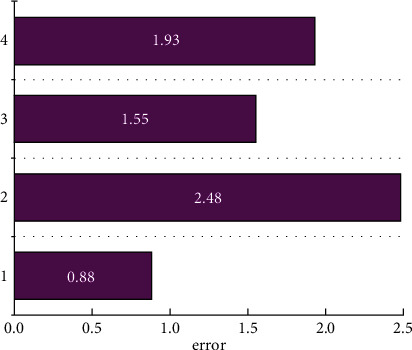
The forecast errors in the sports tourism industry.

**Figure 5:**
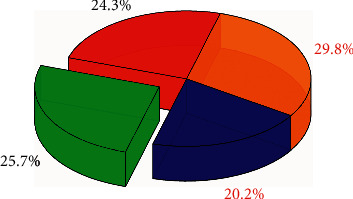
The distribution of knowledge discovery classifications for sports tourism in China.

First, the types of local natural scenery collected in the Chinese sports tourism industry, the participation rate of the historical sports tourism industry, the local economic level, the benefits of the sports tourism industry are classified through the clustering in the knowledge discovery method, so that the next step is the neural network. The network provides data source support.  Figure 4 shows the prediction error of neural network technology for the Chinese sports tourism industry. It can be seen that the prediction errors for the four types of data sources are all within 3%, the largest error is only 2.48%, and the smallest error is only 0.88%. This is an extremely accurate result for a forecast of the sports tourism industry. The biggest error comes from the error of the participation rate, which is mainly due to the obvious time correlation between this variable and the level of local economic development and time. The change in policy has led to a large error, but this error is also more accurate for the prediction of Chinese sports tourism industry. It is more accurate for the prediction of the Chinese sports tourism industry.  Figure 5 shows the classification effect of knowledge discovery methods on variables related to the Chinese sports tourism industry. It can be clearly seen from Figure 5 that the knowledge discovery method can well classify the collected information such as the participation rate of the sports tourism industry and the benefits of the sports tourism industry, and the distribution of the four variables is relatively uniform. It is extremely beneficial for the prediction of the next neural network. The largest proportion is 29.8%, and the smallest proportion is 20.2%. Compared with the imbalanced classification results, this method is more accurate.

**Figure 6:**
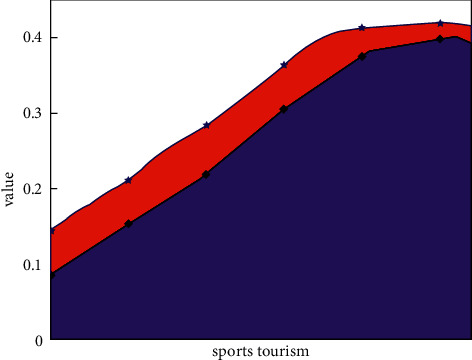
The predicted and actual data sources for the sports tourism industry.

**Figure 7:**
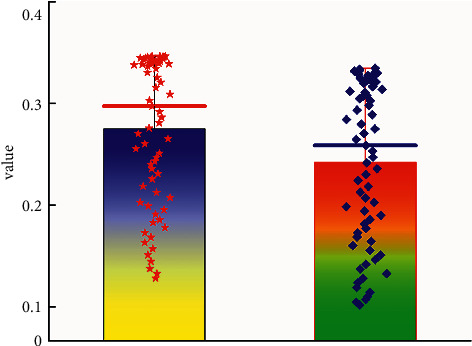
Predicted box value and actual data box plot of the sports tourism industry.

Figure 6 shows the predicted value of the Chinese sports tourism industry predicted by the neural network method and the value of the actual collected data source. It can be clearly seen that the data for the prediction value of sports tourism types are well matched to the actual data sources collected. Their trends over time also correspond well with actual trends. This shows that the ConvLSTM neural network has obvious advantages in predicting the Chinese sports tourism industry. It can not only effectively extract the mapping relationship between data but also reflect the changing trend between different data sources. At the same time, it can be seen intuitively from Figure 6 that the prediction error is in good agreement with the results in Figure 4. In general, the predicted value for the Chinese sports tourism industry is smaller than the actual data source data. This shows that the ConvLSTM neural network model conservatively estimates the value of the sports tourism industry.  Figure 7 shows the box plot of the predicted value of the Chinese sports tourism industry as well as the box plot of the actual collected data. It can be clearly seen that different data sources are in good agreement with the actual data values, and the difference between the mean of the overall data and the mean of the predicted data is relatively small. The distribution of the predicted data values is also in good agreement with the actually collected data sources of the sports tourism industry, which can generally prove that the neural network model is suitable for predicting the relevant data of the Chinese sports tourism industry.

**Figure 8:**
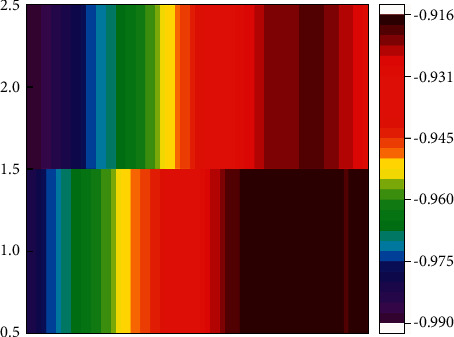
Thermal distribution map of sports tourism industry forecast.

**Figure 9:**
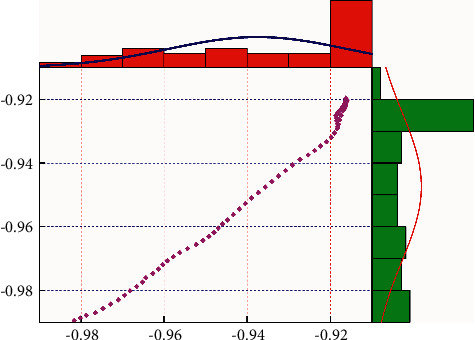
The relevance distribution map of the sports tourism industry.

Figure 8 shows the predicted heat distribution map of the sports tourism industry and the heat distribution map of the actual data source. It can be clearly seen that their distributions are similar, and the distribution widths between different heat sources are also similar, which is more intuitive. The feasibility and accuracy of neural network technology are demonstrated for sports tourism forecasting. However, there is a certain difference between the predicted value and the actual data source on the time axis, which is mainly due to the influence of the iteration of the model, which does not affect the accuracy of sports tourism prediction. There is only a certain width difference at the beginning of the heatmap, which is caused by differences in data sources with strong temporal correlations.  Figure 9 shows the distribution plot of the linear correlation between the predicted value of sports tourism and the actual number of the collection data source. It can be seen that the data points are mainly distributed on both sides of the y = x function, and the distance between the data points and the straight line is relatively close. This can prove that the predicted sports tourism data have a strong correlation with the actual data source. The correlation can reach 0.98. To further verify the generalization ability of the model, a single convolutional neural network without LSTM and other types of tourism data are selected in this paper. It can be seen from Table 2 that the maximum prediction error of a single convolutional neural network without an LSTM layer reaches 3.56%, which is worse than the results of the model in this paper.

## 5. The Summary of Knowledge Discovery for China Sports Tourism

China is a big country with large differences in natural scenery. For regions with different longitudes and latitudes, this promotes the development of tourism. With the improvement of the national economy and the pursuit of sports health, the sports tourism industry has developed rapidly in China. However, for different regions and different sports tourism projects, there are large differences in participation rates. The knowledge discovery method can find certain correlations between different data sources, which provides a certain reference value for the organizers of sports tourism.

At present, the research on sports tourism projects mainly uses algorithms such as decision tree and support vector machine to classify sports tourism projects, which cannot produce a certain reference value for the initiators of sports tourism projects. This paper not only classifies sports tourism projects but also uses neural network technology to classify and predict the sports tourism industry. This will not only classify current sports but also make predictions about future sports tourism. This study firstly used the advantages of knowledge discovery technology in mining the correlation between data to cluster the collected information such as sports tourism participation rate, sports tourism type, and local residents' support rate and then used neural network technology to analyze the sports tourism industry. Forecasts of future trends are made. In general, knowledge discovery technology is suitable for the classification of the sports tourism industry, its classification is relatively uniform, and the error is relatively low. The largest error is 2.48%, and the smallest error is only 0.88%, which is a relatively accurate error range for the prediction of the sports tourism industry. At the same time, the predicted value of the sports tourism industry is in good agreement with the range and change trend of the actual data source, and the correlation coefficient even reaches 0.98. In general, both the knowledge discovery method and the neural network technology have good accuracy for the prediction of China's sports tourism industry, and it is a relatively feasible method. The model in this paper is compared with the results of a single convolutional neural network without LSTM, and the maximum error reaches 3.56%, which is significantly higher than the data model used in this paper. At the same time, in order to verify the generalization ability of the model, this paper has verified the sports tourism data in other regions, and it can be found that the prediction errors are all within 3%. The model used in this paper can not only extract the spatial characteristics of sports tourism but also extract the temporal characteristics, which is not the ability of other research models.

## Figures and Tables

**Table 1:** Input and output of sports tourism industry.

Input	Output
Passenger participation volume	Passenger participation volume
The local tourism economic	
The type of tourism projects	

**Table 2:** The verification of generalization capability of deep learning models.

Model	Error (%)
ConvLSTM	2.48
CNN without LSTM	3.56

## Data Availability

The data used in this article can be reasonably requested by readers and researchers.
